# Supplementary material for: Influence of Aliphatic Chain Length on Structural, Thermal and Electrochemical Properties of n-alkylene Benzyl Alcohols: A Study of the Odd–Even Effect
Source: Molecules. 2022 Jun 12;27(12):3781. doi: 10.3390/molecules27123781 (PMC9229344; doi:10.3390/molecules27123781)
Supplement: Supplementary file 1 [file molecules-27-03781-s001.zip › molecules-1724352-supplementary.pdf]

molecules

Electronic Supplementary Information

Influence of Aliphatic Chain Length on Structural, Thermal and Electrochemical Properties of *n*-alkylene Benzyl Alcohols: A Study of the Odd–Even Effect

Tomislav Balić [1], Marija Paurević [1], Marta Počkaj [2], Martina Medvidović-Kosanović [1,*], Dominik Goman [1], Aleksandar Széchenyi [1], Zsolt Preisz [3,4] and Sándor Kunsági-Máté [3,5]

[1] Department of Chemistry, Josip Juraj Strossmayer University of Osijek, Cara Hadrijana 8/A, 31000 Osijek, Croatia; tombalic@kemija.unios.hr (T.B.); mstivo@kemija.unios.hr (M.P.); dgoman@kemija.unios.hr (D.G.); szealex@kemija.unios.hr (A.S.)
[2] Faculty of Chemistry and Chemical Technology, University of Ljubljana, Večna pot 113, 1000 Ljubljana, Slovenia; marta.pockaj@fkkt.uni-lj.si
[3] Faculty of Pharmacy, Institute of Organic and Medicinal Chemistry, University of Pécs, Szigeti 12, 7624 Pécs, Hungary; preisz.zsolt@gmail.com (Z.P.); kunsagi-mate.sandor@gytk.pte.hu (S.K.-M.)
[4] Department of Physical Chemistry and Materials Science, Faculty of Sciences, University of Pécs, Ifjúság 6, 7624 Pécs, Hungary
[5] János Szentágothai Research Center, University of Pécs, Ifjúság 20, 7624 Pécs, Hungary
* Correspondence: mmkosano@kemija.unios.hr; Tel.: +385-31-399-959; Fax: +385-31-399-969

Contents

Figure S1. Structure overlay of **Do6OH I** conformer A (red) and B (green). 4

Figure S2. Structure overlay of **Do6OH I** conformer A (red) and **Do6OH II** (blue). 4

Table S1. Crystallographic data and structure refinement details for **Do4OH**, **Do5OH**, **Do6OH I** and **Do6OH II.** 5

Scheme S1. Synthetic route for the preparation of compounds with NMR numbering scheme. 6

Synthesis procedure 6

Preparation of 1,2-bis[2-(hydroxymethyl)phenoxy]butylene (**Do4OH**) 6

Preparation of 1,2-bis[2-(hydroxymethyl)phenoxy]pentylene (**Do5OH**) 6

Preparation of 1,2-bis[2-(hydroxymethyl)phenoxy]hexylene (**Do6OH**) 6

Table S2. Selected interatomic bond distances (Å), valence angles (°) and torsion angles (°) for **Do4OH**, **Do5OH**, **Do6OH I** and **Do6OH II** 6

Figure S3. Hirshfeld surfaces for **Do4OH**, **Do5OH**, **Do6OH I** and **Do6OH II** plotted over d_{norm}. In case of **Do4OH** and **Do6OH I**, two symmetry independent molecules are present in the structure and hence HS of both are depicted 10

Figure S4. The full 2D fingerprint plots of (a) **Do4OH**, (b) **Do5OH**, (c) **Do6OH I** and (d) **Do6OH II**. 11

Figure S5. TG (black) and DSC (red) curves of **Do4OH.** 12

Figure S6. TG (black) and DSC (red) curves of **Do5OH.** 12

Table S3. Thermodynamic data of the DSC measurements for **Do4OH**, **Do5OH** and **Do6OH (Forms I** and **II).** 12

Figure S7. Linear Gibbs free energy functions for **Forms I** and **II.** 13

Figure S8. IR spectrum of **Do4OH**. 14

Figure S9. IR spectrum of **Do5OH**. 15

Fig. S10. IR spectrum of **Do6OH I** 16

Fig. S11. IR spectrum of **Do6OH II** 17

Fig. S12. Spectra overlay of **Do6OH I** (black) and **II** (red) 18

Fig. S13. ^{1}H NMR spectra of **Do4OH** 19

Fig. S14. ^{1}H NMR spectra of **Do5OH** 20

Fig. S15. ^{1}H NMR spectra of **Do6OH** 21

Table S4. Molecular pairs interaction energies (kJ/mol) for **Do4OH**. 22

Table S5. Molecular pairs interaction energies (kJ/mol) for **Do5OH**. 23

Table S6. Molecular pairs interaction energies (kJ/mol) for **Do6OH I**. 24

Table S7. Molecular pairs interaction energies (kJ/mol) for **Do6OH II**... 25

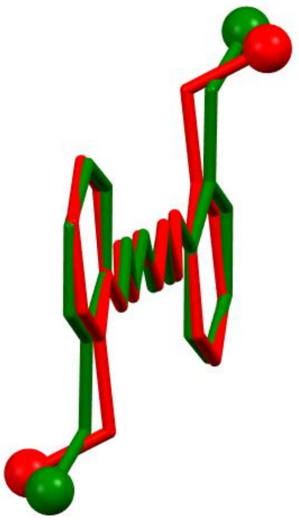

Figure S1. Structure overlay of **Do6OH I** conformer A (red) and B (green).

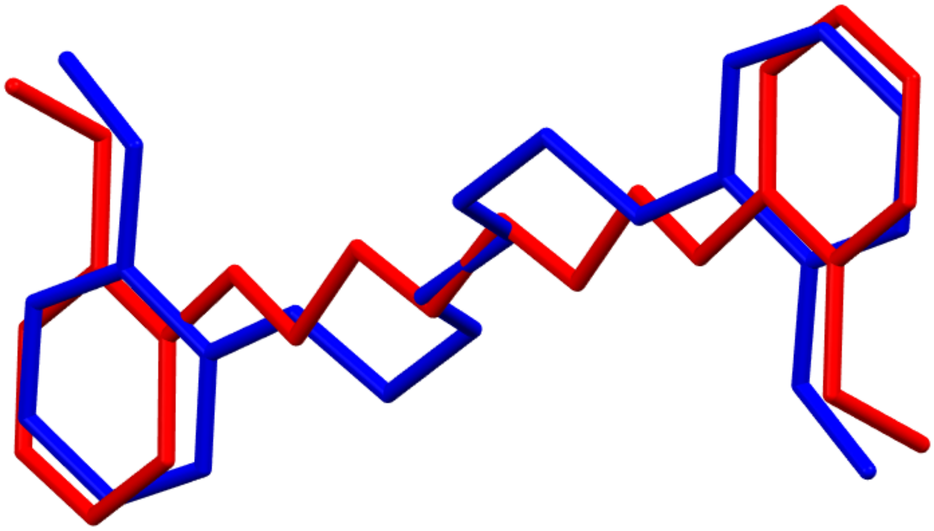

Figure S2. Structure overlay of **Do6OH I** conformer A (red) and **Do6OH II** (blue).

Table S1. Crystallographic data and structure refinement details for **Do4OH**, **Do5OH**, **Do6OH I** and **Do6OH II.**

Identification code	Do4OH	Do5OH	Do6OH I	Do6OH II
Empirical formula	$C_{18}H_{22}O_4$	$C_{19}H_{24}O_4$	$C_{20}H_{26}O_4$	$C_{20}H_{26}O_4$
Formula weight	302.35	316.38	330.41	330.41
Temperature/K	150.15	150.15	150.15	150.15
Crystal system	monoclinic	orthorhombic	monoclinic	orthorhombic
Space group	*I* 2/*a*	*P bcn*	*P* 2_1/*c*	*P bca*
a/Å	25.2277(11)	24.912(4)	11.9940(19)	4.6281(6)
b/Å	5.0156(2)	4.7195(4)	20.4401(18)	14.3917(18)
c/Å	25.7097(13)	14.0976(13)	7.5411(8)	26.534(4)
α/°	90	90	90	90
β/°	92.351(4)	90	91.629(14)	90
γ/°	90	90	90	90
Volume/Å^3	3250.4(3)	1657.5(3)	1848.0(4)	1767.3(4)
Z	8	4	4	4
ρ_{calc}g/cm^3	1.236	1.268	1.188	1.242
μ/mm^{-1}	0.086	0.088	0.081	0.085
F(000)	1296.0	680.0	712.0	712.0
Crystal size/mm^3	0.35 × 0.15 × 0.05	0.3 × 0.15 × 0.1	0.4 × 0.25 × 0.15	0.4 × 0.1 × 0.1
Radiation	Mo Kα (λ = 0.71073)	Mo Kα (λ = 0.71073)	Mo Kα (λ = 0.71073)	Mo Kα (λ = 0.71073)
2Θ range for data collection/°	6.344 to 60.614	5.78 to 60.368	5.238 to 60.61	5.662 to 60.944
Index ranges	-34 ≤ h ≤ 35, -6 ≤ k ≤ 7, -30 ≤ l ≤ 36	-29 ≤ h ≤ 34, -6 ≤ k ≤ 6, -19 ≤ l ≤ 19	-16 ≤ h ≤ 11, -27 ≤ k ≤ 26, -10 ≤ l ≤ 9	-6 ≤ h ≤ 6, -17 ≤ k ≤ 20, -36 ≤ l ≤ 32
Reflections collected	14032	13429	16844	14832
Independent reflections	4405 [R_{int} = 0.0418, R_{sigma} = 0.0580]	2335 [R_{int} = 0.0297, R_{sigma} = 0.0231]	4979 [R_{int} = 0.0285, R_{sigma} = 0.0297]	2525 [R_{int} = 0.0429, R_{sigma} = 0.0320]
Data/restraints/parameters	4405/0/207	2335/0/109	4979/0/225	2525/0/113
Goodness-of-fit on F^2	1.056	1.044	1.034	1.053
Final R indexes [I>=2σ (I)]	R_1 = 0.0695, wR_2 = 0.1874	R_1 = 0.0418, wR_2 = 0.1015	R_1 = 0.0456, wR_2 = 0.1060	R_1 = 0.0457, wR_2 = 0.1072
Final R indexes [all data]	R_1 = 0.1014, wR_2 = 0.2102	R_1 = 0.0545, wR_2 = 0.1112	R_1 = 0.0614, wR_2 = 0.1168	R_1 = 0.0718, wR_2 = 0.1197
Largest diff. peak/hole / e Å^{-3}	0.36/-0.24	0.22/-0.19	0.26/-0.24	0.19/-0.19

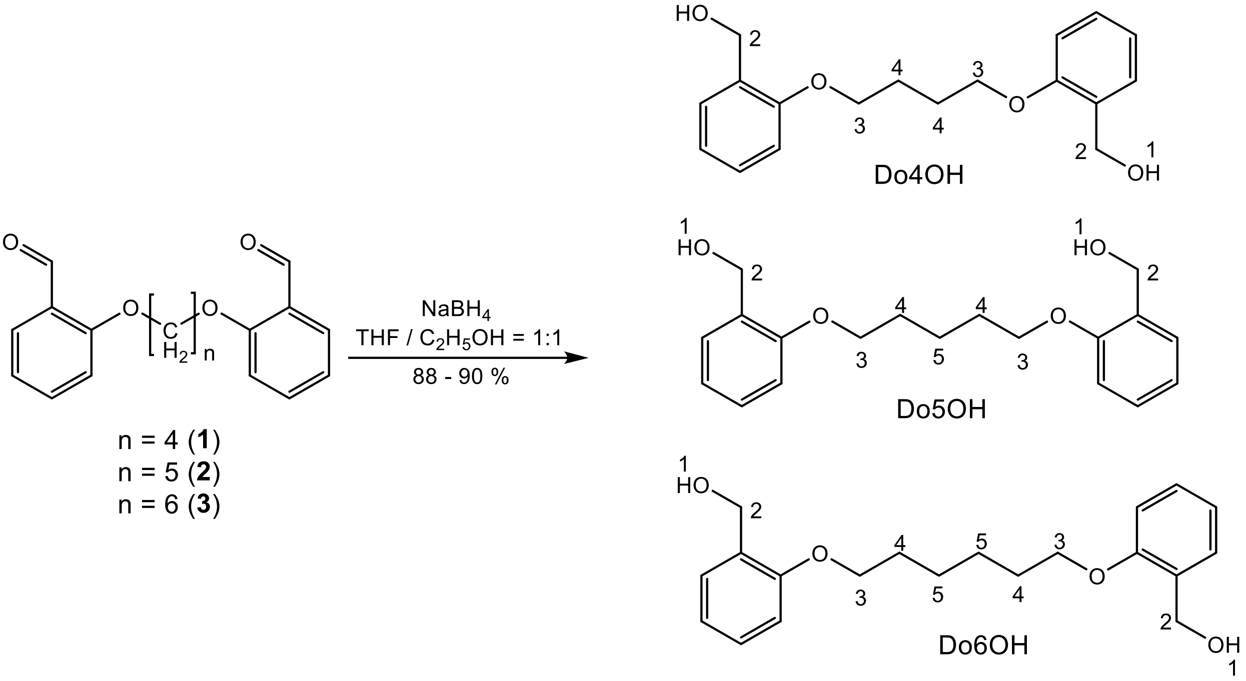

Scheme S1. Synthetic route for the preparation of compounds with NMR numbering scheme.

Synthesis procedure

Preparation of 1,2-bis[2-(hydroxymethyl)phenoxy]butylene (**Do4OH**)

Sodium borohydride (304.4 mg, 8.05 mmol) was slowly added to a THF/C_2H_5OH (15 mL, in 1:1 ratio) solution of dialdehyde **1** (1 g, 3.35 mmol) with stirring for 2.5 hours in the ice-water bath. The reaction mixture was refluxed for 10 hours. The solvent was then evaporated and 15 mL of H_2O was added to the residue. The resulting solution was extracted with CH_2Cl_2 and the organic phase was dried over anhydrous Na_2SO_4. After removing CH_2Cl_2, a white solid of 1,2-bis[2-(hydroxymethyl)phenoxy]butylene was obtained. Yield: 887.5 mg (88 %). ^{1}H NMR (DMSO) δ / ppm: 7.37 (dd, 2H, H-Ar), 6.91-7.19 (m, 6H, H-Ar), 4.94-4.96 (t, 2H, H-1), 4.51 (d, 4H, H-2), 4.03 (t, 4H, H-3), 1.88 (m, 4H, H-4). An identical procedure was used to obtain **Do5OH** and **Do6OH**.

Preparation of 1,2-bis[2-(hydroxymethyl)phenoxy]pentylene (**Do5OH**)

Yield: 900 mg (88 %). ^{1}H NMR (DMSO) δ / ppm: 7.33 (dd, 2H, H-Ar), 7.12 (td, 2H, H-Ar), 6.86-6.88 (m, 4H, H-Ar) 4.90 (t, 2H, H-1), 4.46 (d, 4H, H-2), 3.93 (t, 4H, H-3), 1.74 (quin, 4H, H-4), 1.52-1.57 (m, 2H, H-5).

Preparation of 1,2-bis[2-(hydroxymethyl)phenoxy]hexylene (**Do6OH**)

Yield: 559 mg (90 %). ^{1}H NMR (DMSO) δ / ppm: 7.35-7.37 (dd, 2H, H-Ar), 7.16-7.19 (td, 2H, H-Ar), 6.89-6.92 (m, 4H, H-Ar), 4.93-4.95 (t, 2H, H-1), 4.91-4.50 (d, 4H, H-2), 3.96-3.98 (t, 4H, H-3), 1.73-1.75 (m, 4H, H-4), 1.49 (m, 4H, H-5).

Table S2. Selected interatomic bond distances (Å), valence angles (°) and torsion angles (°) for **Do4OH**, **Do5OH**, **Do6OH I** and **Do6OH II**.

Selected bond distances (Å)		Selected bond angles (°)		Selected torsion angles (°)	
**Do4OH**					
C1-O1	1.426(3)	O1-C1-C2	112.15(19)	O1-C1-C2-C3	-2.6(3)
C1-C2	1.510(3)	C3-C2-C7	118.6(2)	O1-C1-C2-C7	177.15(18)

C2-C3	1.384(3)	C3-C2-C1	122.5(2)	C7-C2-C3-C4	0.7(3)
C2-C7	1.402(3)	C7-C2-C1	118.85(19)	C1-C2-C3-C4	-179.5(2)
C3-C4	1.392(3)	C2-C3-C4	120.7(2)	C2-C3-C4-C	50.2(4)
C4-C5	1.369(4)	C5-C4-C3	120.0(2)	C3-C4-C5-C6	-0.3(4)
C5-C6	1.395(3)	C4-C5-C6	120.7(2)	C4-C5-C6-C7	-0.7(3)
C6-C7	1.385(3)	C7-C6-C5	119.1(2)	C5-C6-C7-O2	-177.22(19)
C7-O2	1.372(3)	O2-C7-C6	124.4(2)	C5-C6-C7-C	21.7(3)
C8-O2	1.431(3)	O2-C7-C2	114.70(19)	C3-C2-C7-O2	177.30(18)
C8-C9	1.505(3)	C6-C7-C2	120.9(2)	C1-C2-C7-O2	-2.5(39
C9-C9#1	1.517(4)	O2-C8-C9	106.78(18)	C3-C2-C7-C6	-1.7(3)
C10-O3	1.425(3)	C8-C9-C9#1	111.7(2)	C1-C2-C7-C6	178.5(2)
C10-C11	1.500(3)	O3-C10-C11	112.68(19)	O2-C8-C9-C9#1	-179.4(2)
C11-C12	1.377(3)	C12-C11-C16	118.1(2)	O3-C10-C11-C12	-0.9(3)
C11-C16	1.413(3)	C12-C11-C10	123.4(2)	O3-C10-C11-C16	177.9(2)
C12-C13	1.382(4)	C16-C11-C10	118.5(2)	C16-C11-C12-C1	31.1(3)
C13-C14	1.387(4)	C11-C12-C13	121.5(2)	C10-C11-C12-C13	179.9(2)
C14-C15	1.382(4)	C12-C13-C14	119.5(2)	C11-C12-C13-C1	40.1(4)
C15-C16	1.379(3)	C15-C14-C13	120.5(2)	C12-C13-C14-C15	-0.5(4)
C16-O4	1.370(3)	C16-C15-C14	119.5(2)	C13-C14-C15-C16	-0.3(4)
C17-O4	1.431(3)	O4-C16-C15	124.9(2)	C14-C15-C16-O4	-178.0(2)
C17-C18	1.509(4)	O4-C16-C11	114.19(19)	C14-C15-C16-C11	1.6(3)
C18-C18#2	1.518(5)	C15-C16-C11	120.9(2)	C12-C11-C16-O4	177.69(19)
		O4-C17-C18	106.76(19)	C10-C11-C16-O4	-1.2(3)
		C17-C18-C18#2	111.7(3)	C12-C11-C16-C15	-1.9(3)
		C1-O1-H1	105.7(18)	C10-C11-C16-C15	179.2(2)
		C7-O2-C8	117.63(17)	O4-C17-C18-C18#2	177.7(2)
		C16-O4-C17	118.04(17)	C6-C7-O2-C8	0.1(3)
				C2-C7-O2-C8	-178.91(18)
				C9-C8-O2-C7	175.43(17)
				C15-C16-O4-C17	1.1(39
				C11-C16-O4-C17	-178.45(19)
				C18-C17-O4-C16	174.46(19)
#1 -x+1,-y+1,-z #2 -x+1/2,-y+5/2,-z+1/2					
**Do5OH**					
C1-O1	1.4239(13)	O1-C1-C2	113.59(10)	O1-C1-C2-C3	14.46(16)
C1-C2	1.5080(16)	C3-C2-C7	118.59(11)	O1-C1-C2-C7	-166.02(10)
C2-C3	1.3897(16)	C3-C2-C1	123.05(11)	C7-C2-C3-C4	-0.28(17)

C2-C7	1.3998(16)	C7-C2-C1	118.36(10)	C1-C2-C3-C4	179.24(11)
C3-C4	1.3921(18)	C2-C3-C4	121.20(12)	C2-C3-C4-C5	0.68(18)
C4-C5	1.3788(19)	C5-C4-C3	119.32(11)	C3-C4-C5-C6	0.04(18)
C5-C6	1.3917(17)	C4-C5-C6	120.92(12)	C4-C5-C6-C7	-1.15(17)
C6-C7	1.3920(16)	C5-C6-C7	119.26(12)	C5-C6-C7-O2	-178.30(10)
C7-O2	1.3769(13)	O2-C7-C6	124.40(10)	C5-C6-C7-C2	1.55(16)
C8-O2	1.4314(13)	O2-C7-C2	114.90(10)	C3-C2-C7-O2	179.01(9)
C8-C9	1.5102(16)	C6-C7-C2	120.70(10)	C1-C2-C7-O2	-0.52(14)
C9-C10	1.5240(14)	O2-C8-C9	107.99(9)	C3-C2-C7-C6	-0.85(16)
C10-C9#1	1.5240(13)	C8-C9-C10	114.17(9)	C1-C2-C7-C6	179.62(10)
		C9#1-C10-C9	112.26(12)	O2-C8-C9-C10	-62.65(11)
		C7-O2-C8	117.42(9)	C8-C9-C10-C9#1	-179.50(11)
				C6-C7-O2-C8	-0.63(15)
				C2-C7-O2-C8	179.52(9)
				C9-C8-O2-C7	179.89(8)
#1 -x+1,y,-z+3/2					
**Do6OH I**					
C1-O1	1.4276(15)	O1-C1-C2	112.66(11)	O1-C1-C2-C3	87.50(14)
C1-C2	1.5021(18)	C3-C2-C7	118.58(12)	O1-C1-C2-C7	-91.84(13)
C2-C3	1.3898(17)	C3-C2-C1	120.86(12)	C7-C2-C3-C4	-0.78(19)
C2-C7	1.4022(17)	C7-C2-C1	120.56(11)	C1-C2-C3-C4	179.87(12)
C3-C4	1.386(2)	C4-C3-C2	121.24(13)	C2-C3-C4-C5	-0.9(2)
C4-C5	1.383(2)	C5-C4-C3	119.43(12)	C3-C4-C5-C6	1.1(2)
C5-C6	1.3928(18)	C4-C5-C6	120.70(13)	C4-C5-C6-C7	0.47(19)
C6-C7	1.3887(17)	C7-C6-C5	119.37(13)	C5-C6-C7-O2	179.16(11)
C7-O2	1.3687(13)	O2-C7-C6	123.82(11)	C5-C6-C7-C2	-2.19(18)
C8-O2	1.4332(14)	O2-C7-C2	115.53(10)	C3-C2-C7-O2	-178.90(10)
C8-C9	1.5054(16)	C6-C7-C2	120.63(11)	C1-C2-C7-O2	0.45(16)
C9-C10	1.5270(16)	O2-C8-C9	108.18(10)	C3-C2-C7-C6	2.34(18)
C10-C10#1	1.522(2)	C8-C9-C10	111.49(10)	C1-C2-C7-C6	-178.31(11)
C11-O3	1.4159(15)	C10#1-C10-C9	112.83(13)	O2-C8-C9-C10	-175.44(10)
C11-C12	1.5070(17)	O3-C11-C12	114.65(10)	C8-C9-C10-C10#1	177.06(12)
C12-C13	1.3848(17)	C13-C12-C17	118.67(11)	O3-C11-C12-C13	-10.45(18)
C12-C17	1.4039(16)	C13-C12-C11	123.55(11)	O3-C11-C12-C17	168.46(11)
C13-C14	1.3906(18)	C17-C12-C11	117.77(10)	C17-C12-C13-C14	-0.44(19)
C14-C15	1.3772(18)	C12-C13-C14	121.17(12)	C11-C12-C13-C14	178.46(12)
C15-C16	1.3917(17)	C15-C14-C13	119.38(12)	C12-C13-C14-C15	-0.2(2)

C16-C17	1.3888(17)	C14-C15-C16	120.94(12)	C13-C14-C15-C16	0.8(2)
C17-O4	1.3675(14)	C17-C16-C15	119.24(11)	C14-C15-C16-C17	-0.88(19)
C18-O4	1.4339(14)	O4-C17-C16	124.80(10)	C15-C16-C17-O4	-179.33(11)
C18-C19	1.5056(17)	O4-C17-C12	114.61(10)	C15-C16-C17-C12	0.25(18)
C19-C20	1.5222(16)	C16-C17-C12	120.59(11)	C13-C12-C17-O4	-179.98(11)
C20-C20#2	1.524(2)	O4-C18-C19	107.55(10)	C11-C12-C17-O4	1.05(16)
		C18-C19-C20	112.34(11)	C13-C12-C17-C16	0.39(18)
		C19-C20-C20#2	112.51(13)	C11-C12-C17-C16	-178.57(11)
		C7-O2-C8	118.08(9)	O4-C18-C19-C20	177.05(10)
		C17-O4-C18	118.37(9)	C18-C19-C20-C20#2	-177.45(13)
				C6-C7-O2-C8	-7.89(17)
				C2-C7-O2-C8	173.39(10)
				C9-C8-O2-C7	-176.53(10)
				C16-C17-O4-C18	10.10(17)
				C12-C17-O4-C18	-169.51(10)
				C19-C18-O4-C17	172.67(10)
#1 -x,-y,-z+2 #2 -x+1,-y,-z+1					
**Do6OH 2**					
C1-O1	1.4182(16)	O1-C1-C2	113.78(12)	O1-C1-C2-C3	17.9(2)
C1-C2	1.500(2)	C3-C2-C7	118.34(13)	O1-C1-C2-C7	-162.92(12)
C2-C3	1.3827(19)	C3-C2-C1	122.45(13)	C7-C2-C3-C4	0.1(2)
C2-C7	1.3934(19)	C7-C2-C1	119.20(12)	C1-C2-C3-C4	179.29(14)
C3-C4	1.381(2)	C4-C3-C2	121.17(15)	C2-C3-C4-C5	-0.1(2)
C4-C5	1.374(2)	C5-C4-C3	119.45(14)	C3-C4-C5-C6	0.5(2)
C5-C6	1.385(2)	C4-C5-C6	120.84(15)	C4-C5-C6-C7	-0.9(2)
C6-C7	1.3794(19)	C7-C6-C5	119.09(14)	C5-C6-C7-O2	-178.97(12)
C7-O2	1.3738(15)	O2-C7-C6	123.98(12)	C5-C6-C7-C2	0.9(2)
C8-O2	1.4259(16)	O2-C7-C2	114.92(12)	C3-C2-C7-O2	179.36(11)
C8-C9	1.5015(19)	C6-C7-C2	121.10(12)	C1-C2-C7-O2	0.19(18)
C9-C10	1.5144(18)	O2-C8-C9	107.78(11)	C3-C2-C7-C6	-0.56(19)
C10-C10#1	1.516(3)	C8-C9-C10	114.22(11)	C1-C2-C7-C6	-179.73(13)
		C9-C10-C10#1	113.08(13)	O2-C8-C9-C10	-67.30(14)
		C7-O2-C8	117.65(10)	C8-C9-C10-C10#1	-178.39(14)
				C6-C7-O2-C8	-3.79(18)
				C2-C7-O2-C8	176.29(11)
				C9-C8-O2-C7	-177.73(10)
#1 -x,-y+1,-z+1					

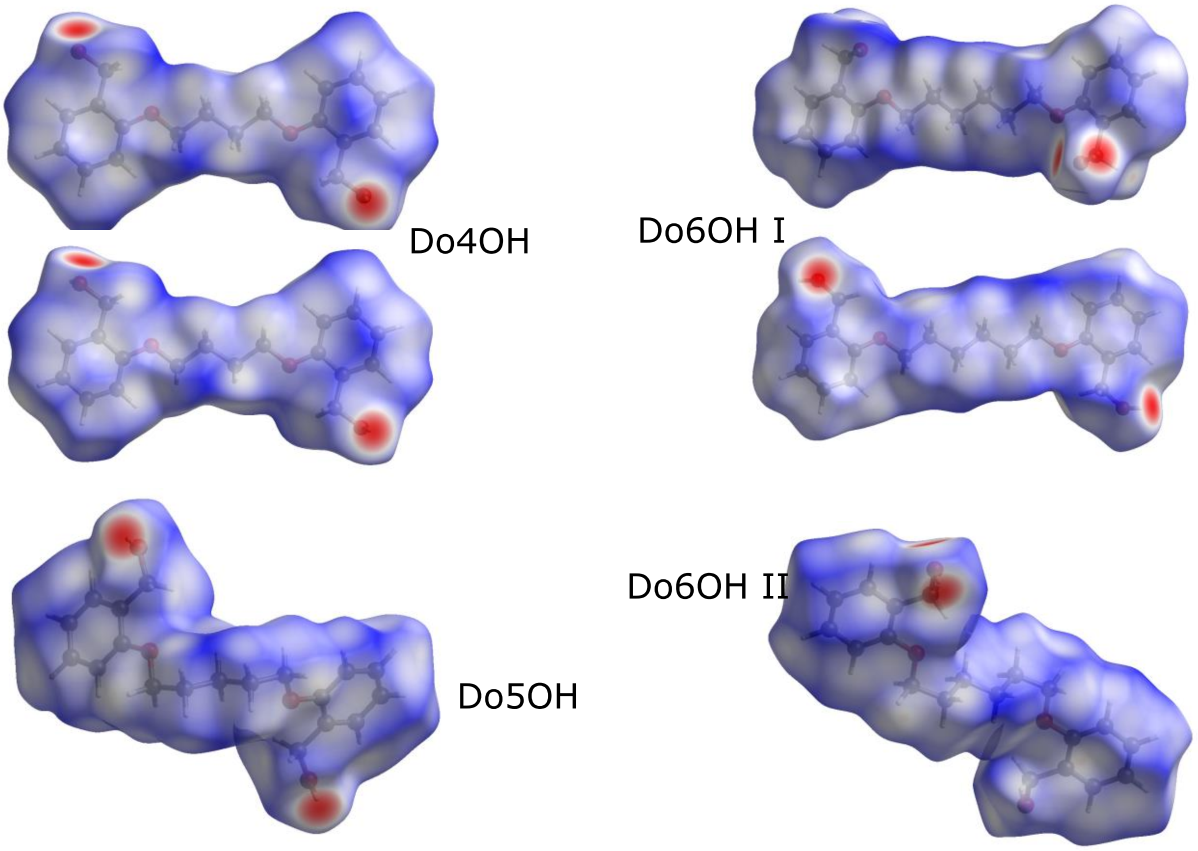

Figure S3. Hirshfeld surfaces for **Do4OH**, **Do5OH**, **Do6OH I** and **Do6OH II** plotted over d_{norm}. In case of **Do4OH** and **Do6OH I**, two symmetry independent molecules are present in the structure and hence HS of both are depicted.

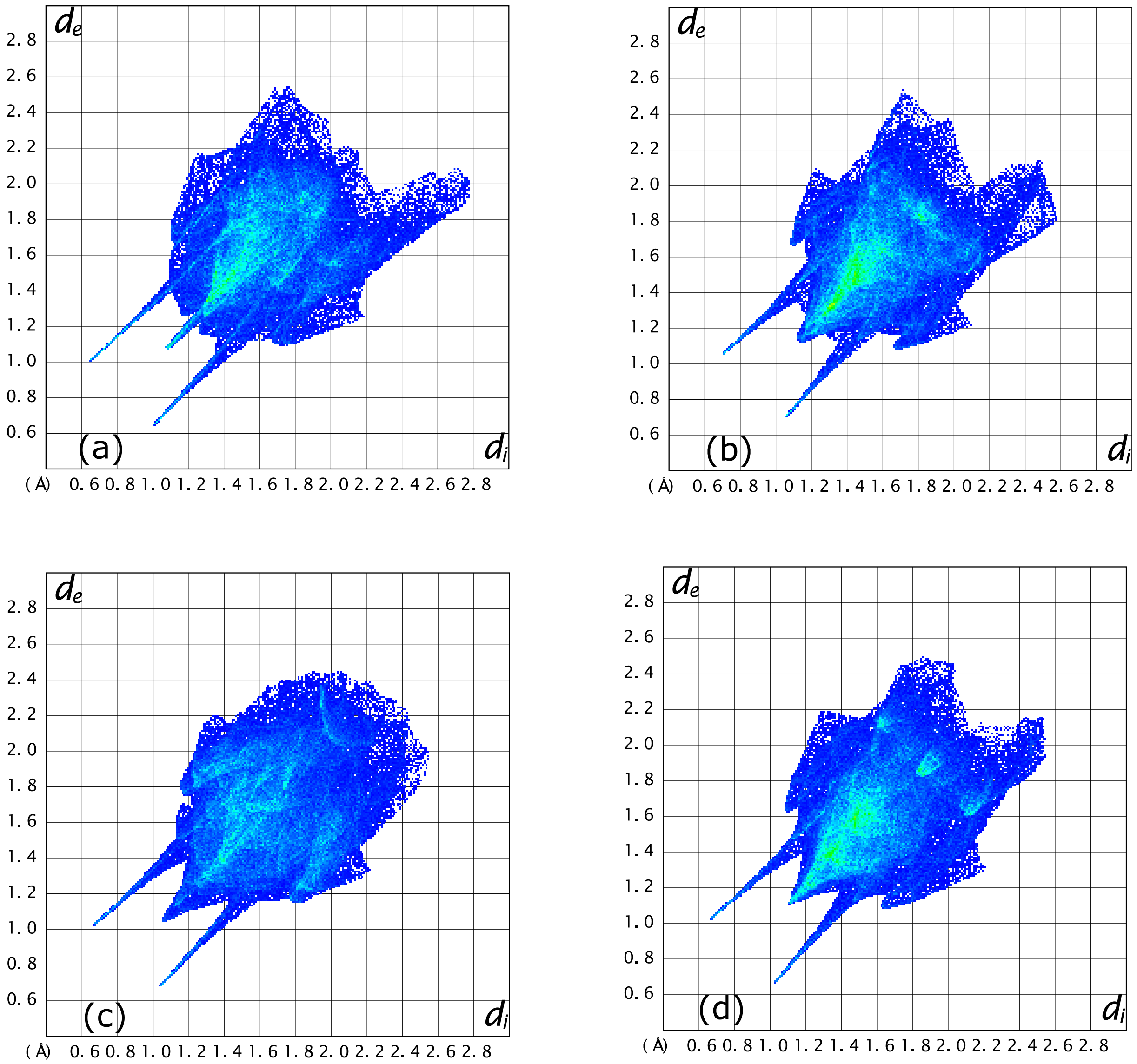

Figure S4. The full 2D fingerprint plots of (a) **Do4OH**, (b) **Do5OH**, (c) **Do6OH I** and (d) **Do6OH II**.

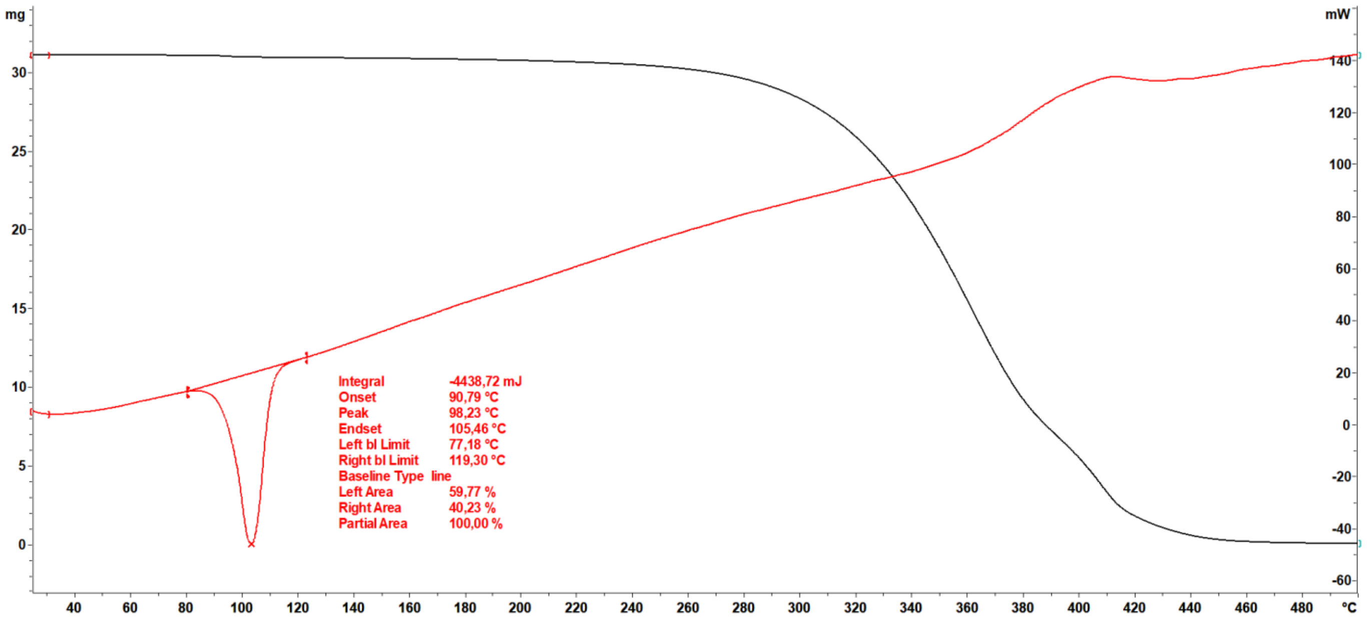

Figure S5. TG (black) and DSC (red) curves of **Do4OH.**

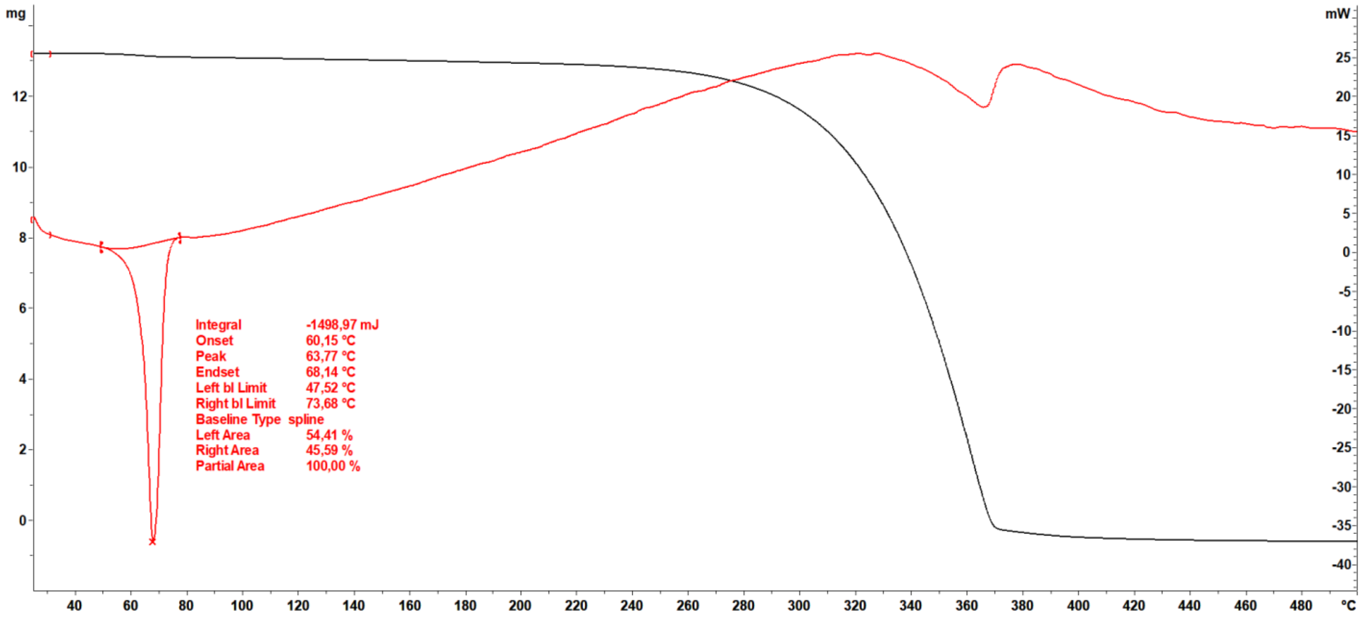

Figure S6. TG (black) and DSC (red) curves of **Do5OH.**

Table S3. Thermodynamic data of the DSC measurements for **Do4OH**, **Do5OH** and **Do6OH (Forms I** and **II).**

Compound	T_{fus}/K	ΔH_{fus}/kJ mol^{-1}	ΔS_{fus} /kJ mol^{-1} K^{-1}
**Do4OH**	371.38	42.6	0.115
**Do5OH**	336.92	37.33	0.111
**Do6OH I** (DMF)	336.09	17.84	0.053
**Do6OH II (DMSO**)	333.07	27.09	0.081

T_{fus} – melting point
ΔH_{fus} – enthalpy of fusion
ΔS_{fus} – entropy of fusion

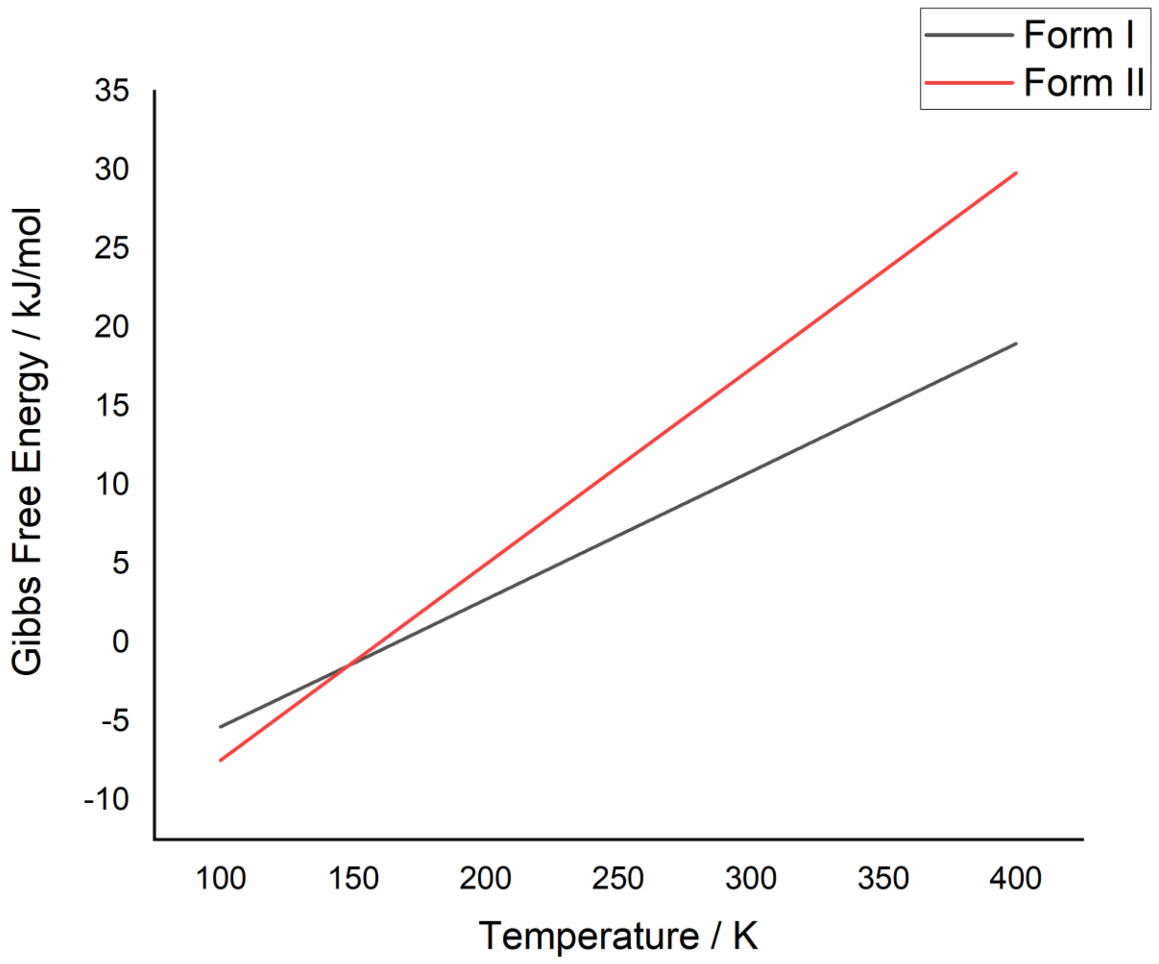

Figure S7. Linear Gibbs free energy functions for **Forms I** and **II.**

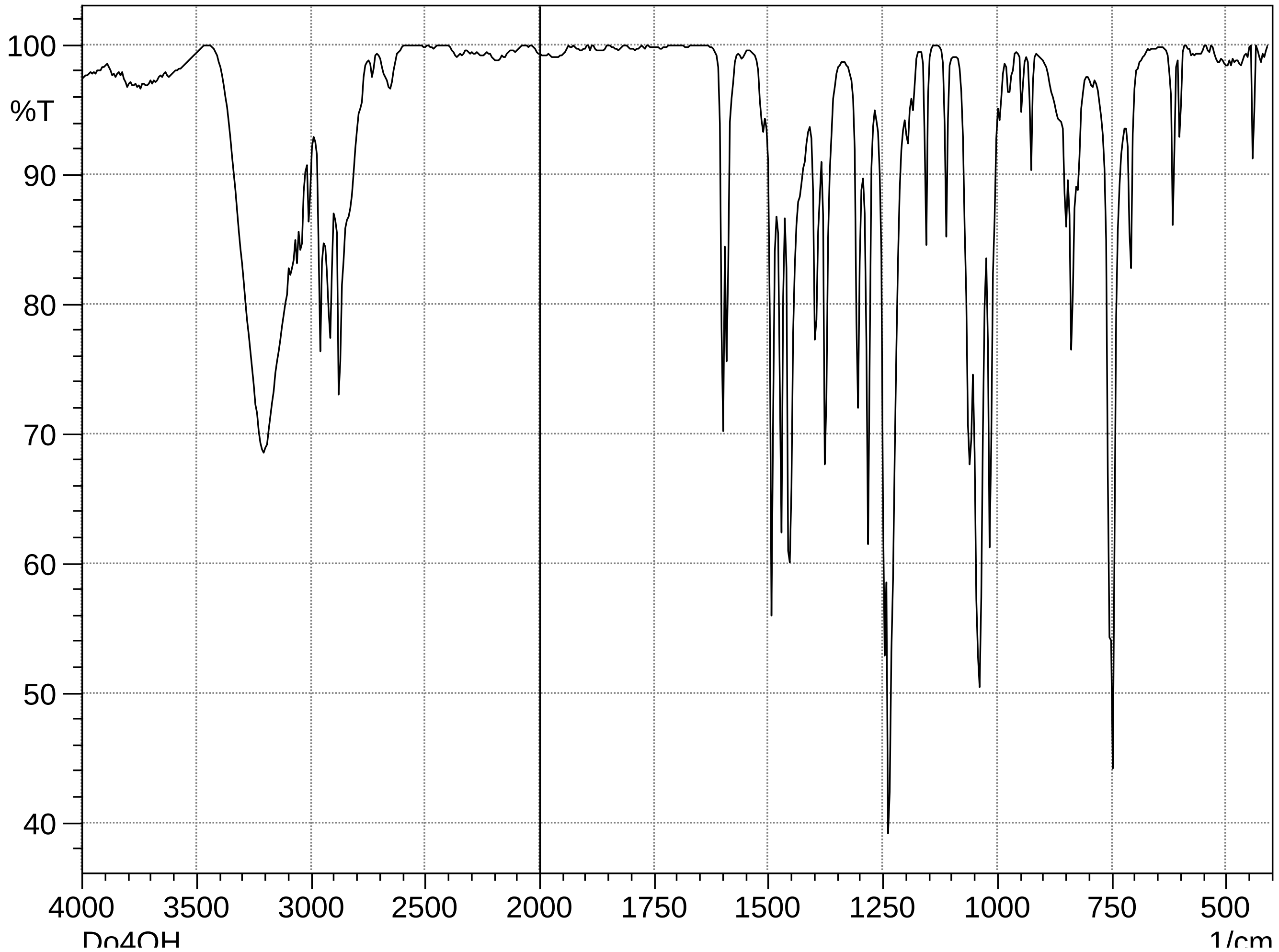

Figure S8. IR spectrum of **Do4OH**.

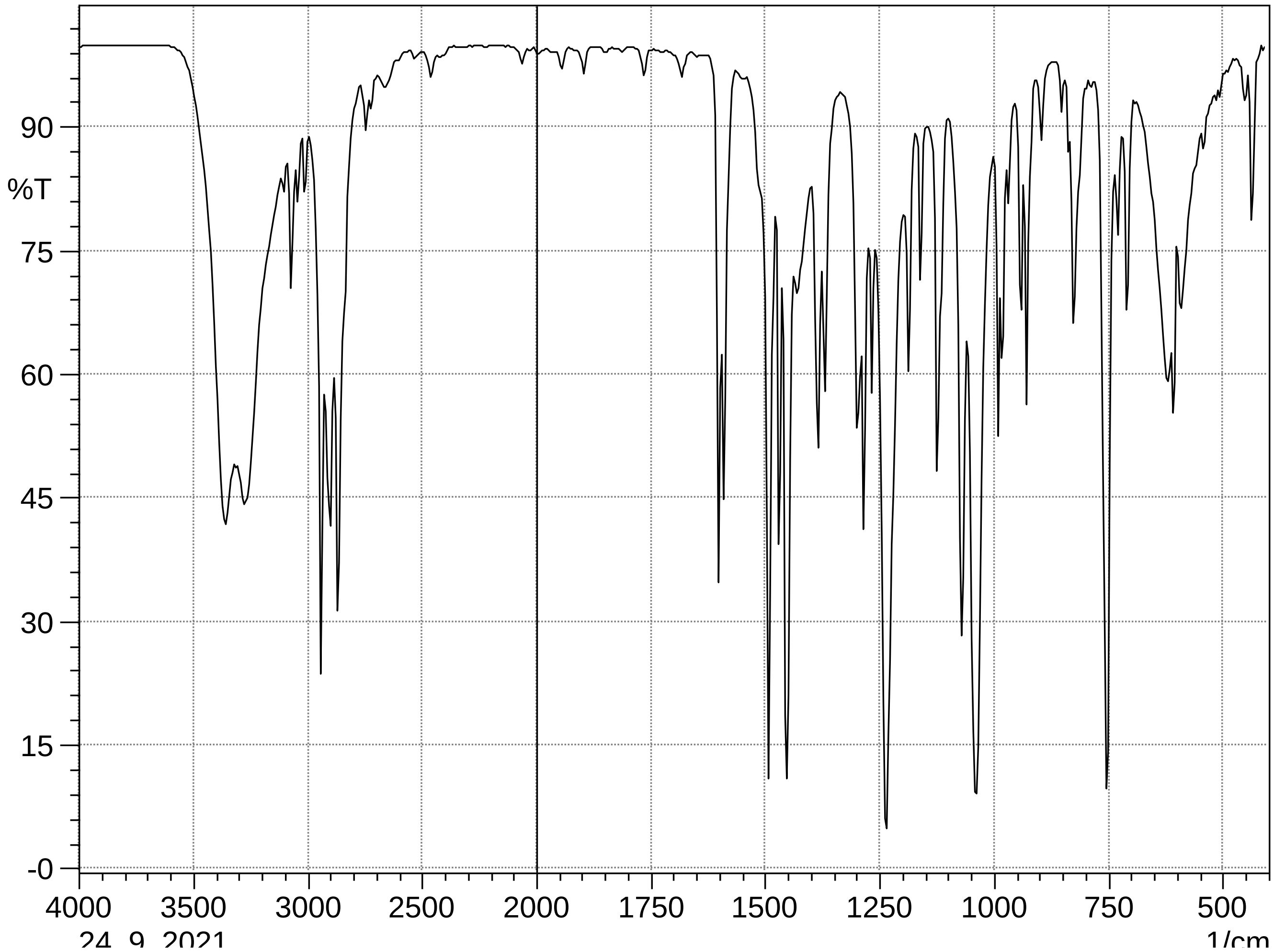

Figure S9. IR spectrum of **Do5OH**.

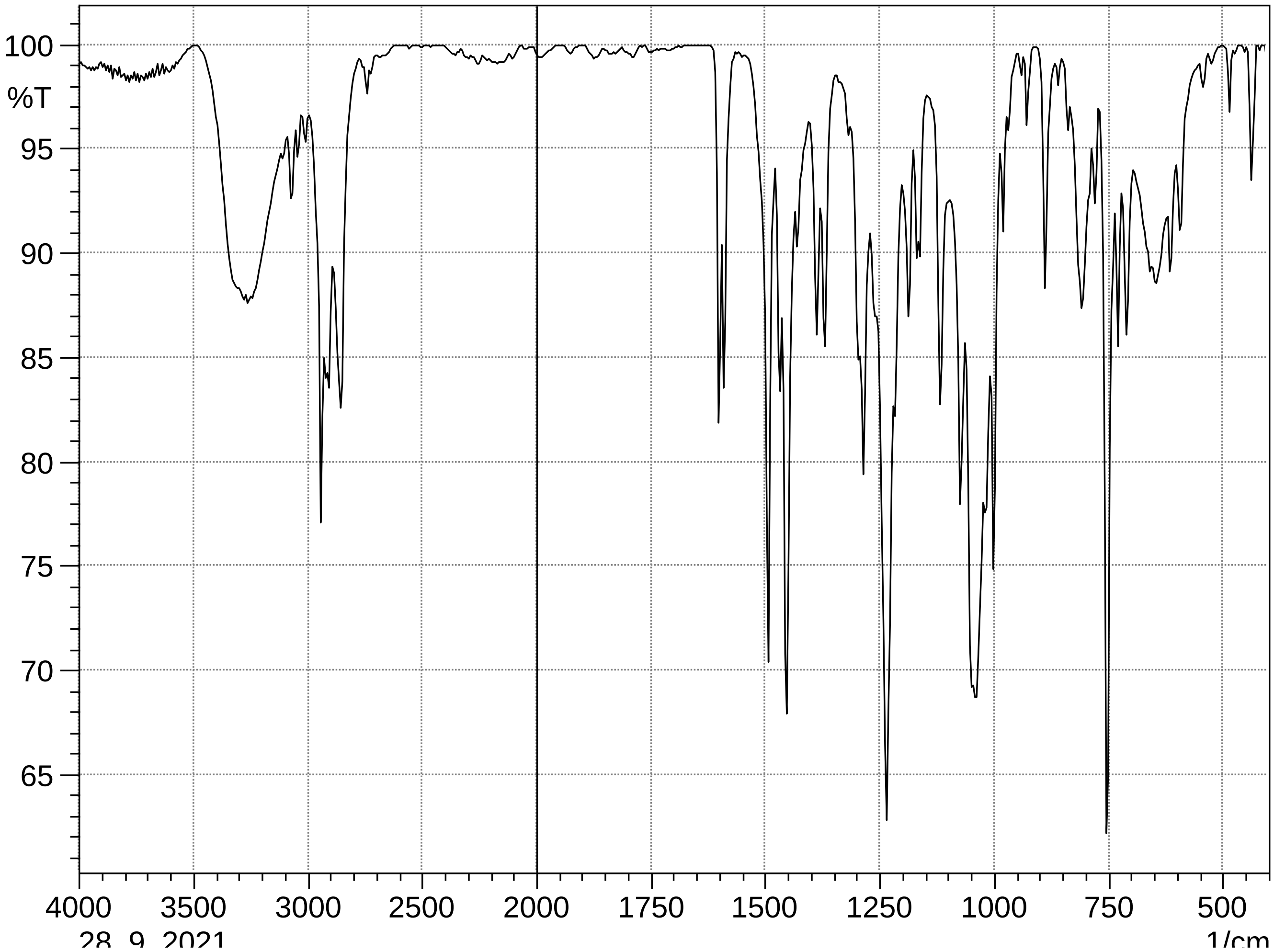

Figure S10. IR spectrum of **Do6OH I**

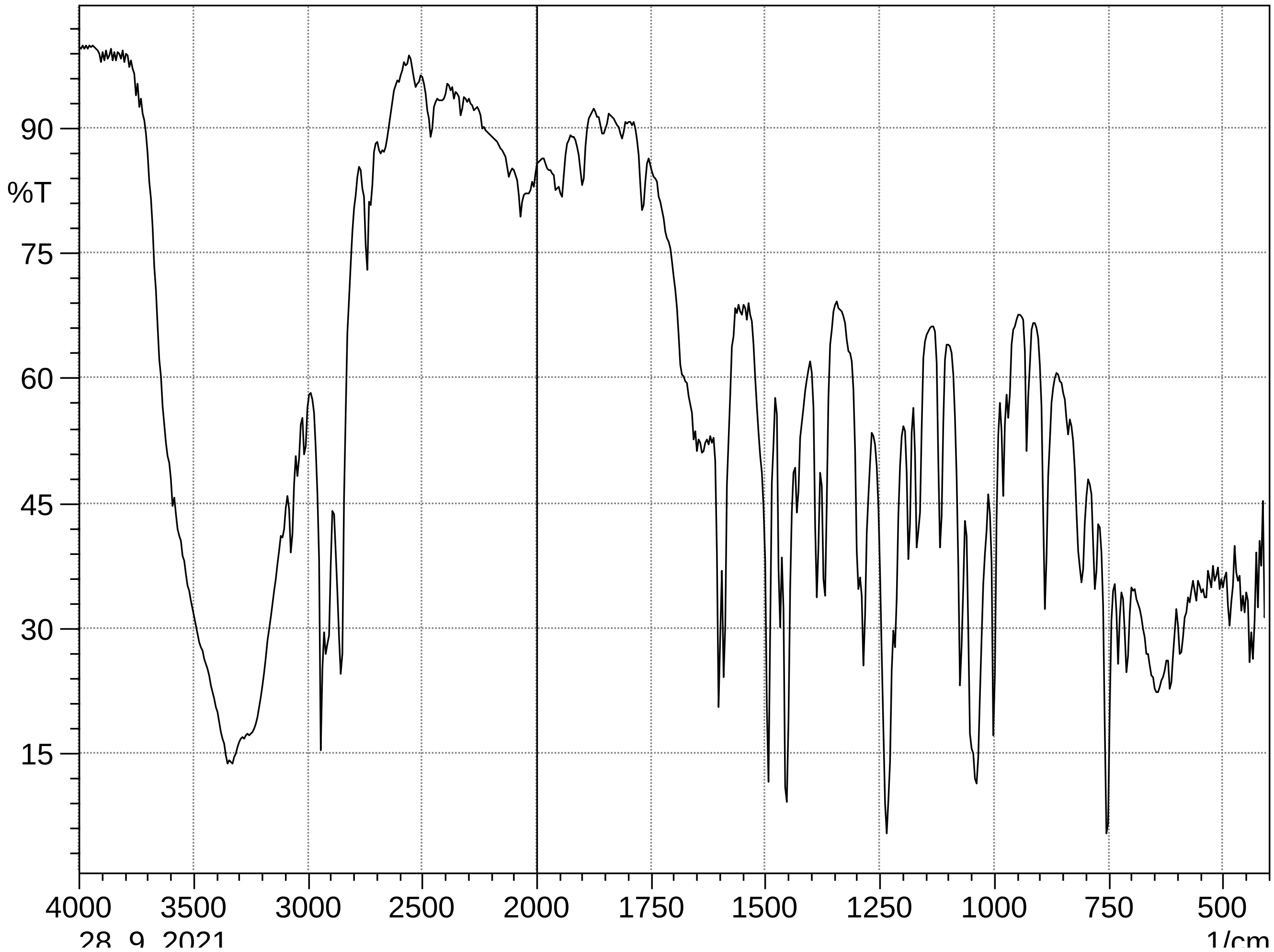

Figure S11. IR spectrum of **Do6OH II**

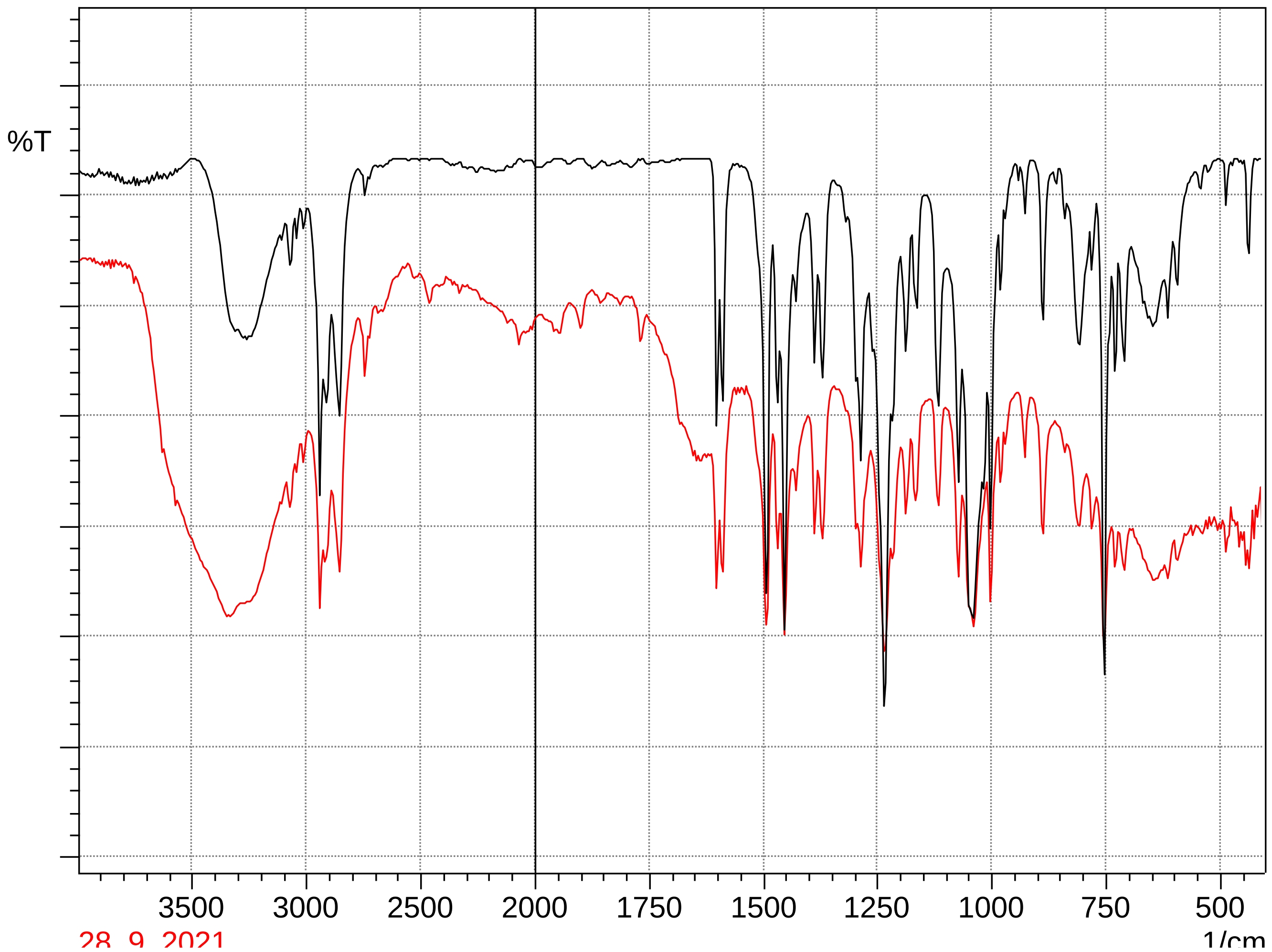

Figure S12. Spectra overlay of **Do6OH I** (black) and **II** (red)

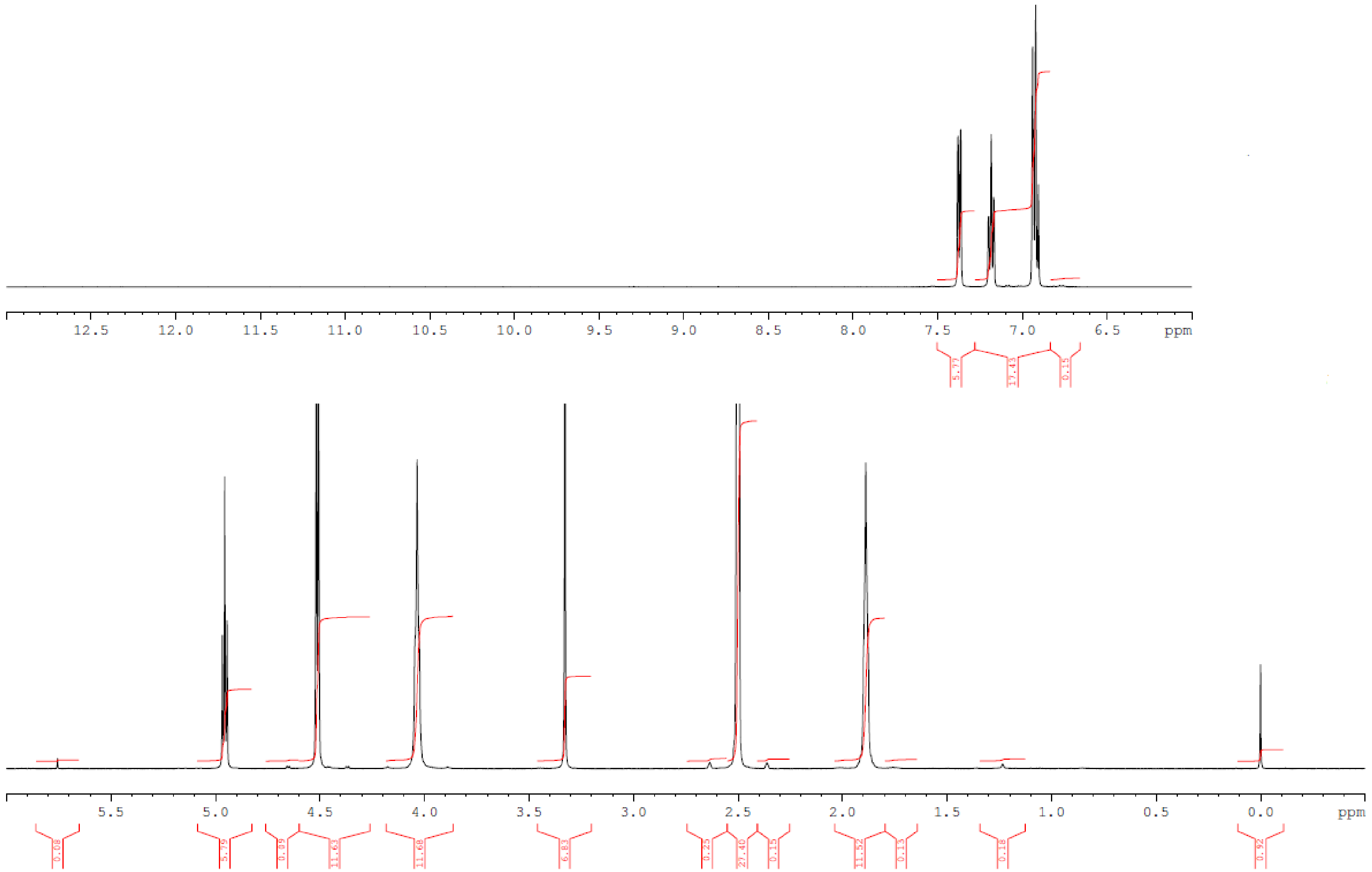

Figure S13. ¹H NMR spectra of **Do4OH**

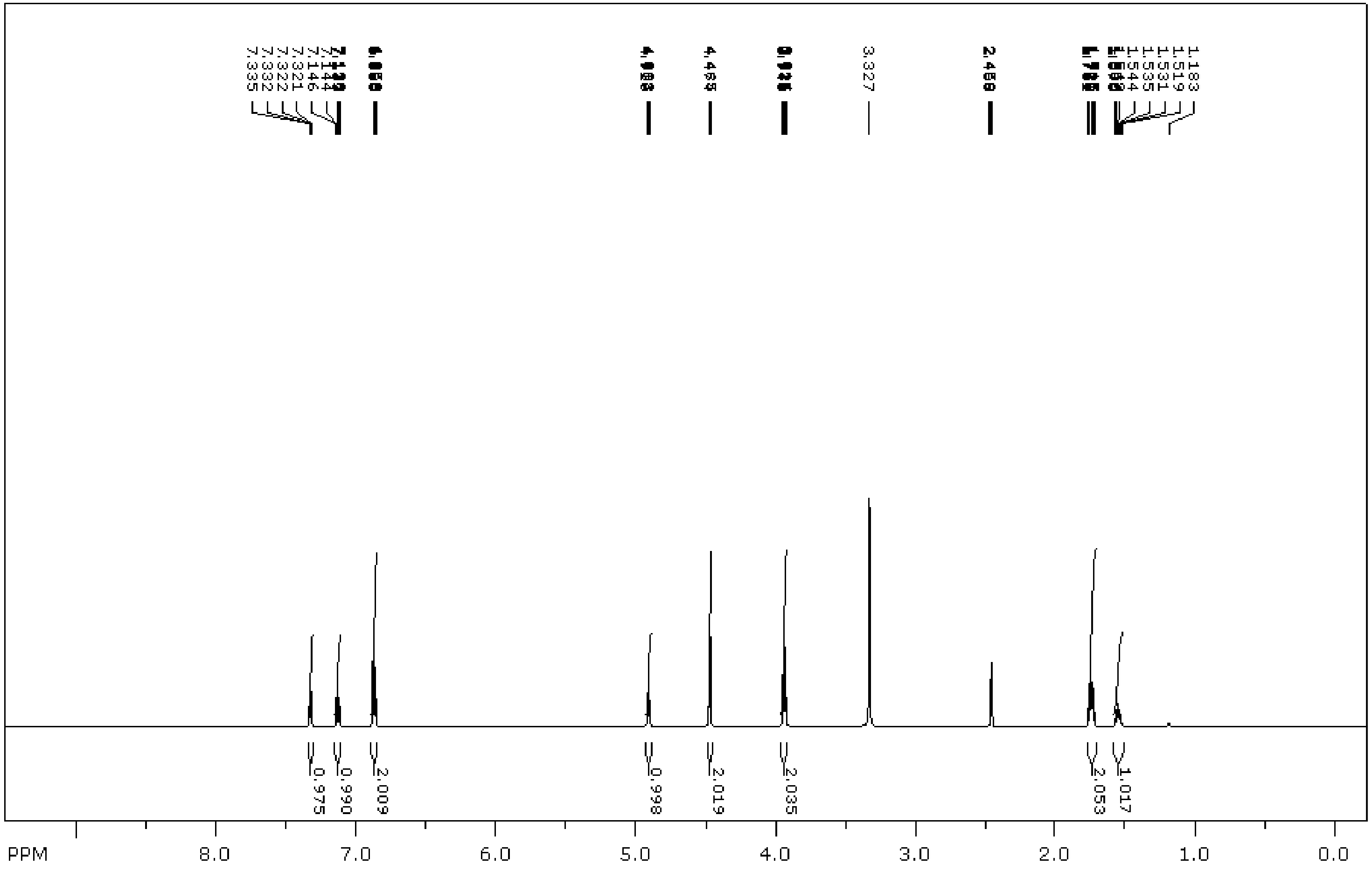

Figure S14. ^{1}H NMR spectra of **Do5OH**

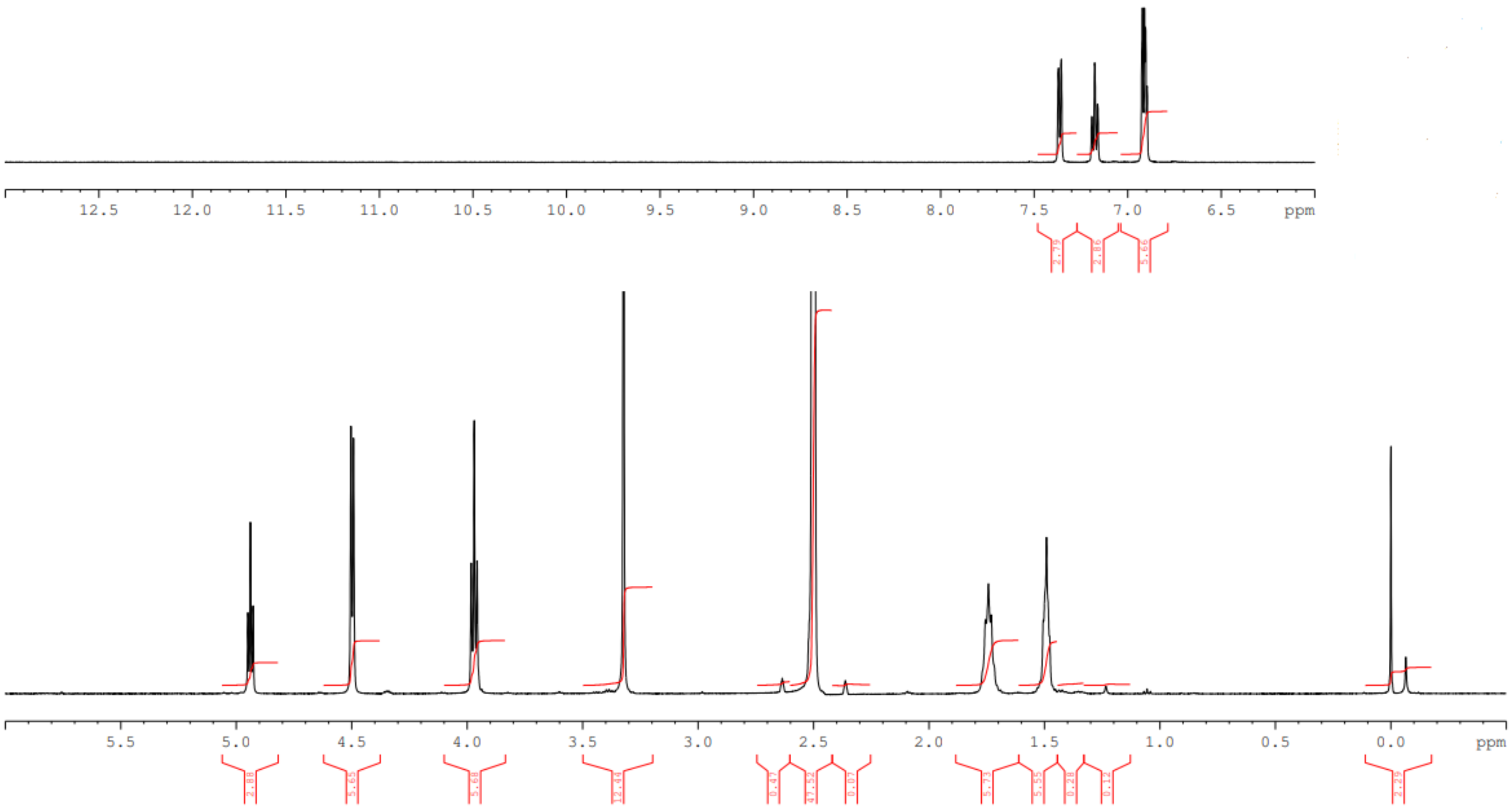

Figure S15. ^{1}H NMR spectra of **Do6OH**

Table S4. Molecular pairs interaction energies (kJ/mol) for **Do4OH**.

N	Symop	R	E_ele	E_pol	E_dis	E_rep	E_tot
2	-	9.93	-3.3824	-0.74	-19.162	6.5508	-16.7336
2	-	9.27	-58.3464	-9.028	-12.6295	46.659	-33.3449
4	-x, y+1/2, -z+1/2	12.86	-2.114	-0.222	-1.8291	0.0618	-4.1033
1	-	12.71	-2.7482	-0.296	-9.8423	4.8822	-8.0043
2	-	8.91	-58.5578	-9.25	-12.5424	47.4624	-32.8878
2	-x+1/2, y, -z	12.85	0.1057	-0.074	-2.1775	0.309	-1.8368
2	-	9.59	-4.9679	-0.888	-20.8169	7.7868	-18.886

1	-	12.44	-2.5368	-0.37	-10.6262	4.8204	-8.7126
2	x, y, z	5.02	-17.6519	-3.7	-72.3801	30.1584	-63.5736
2	-x+1/2, y, -z	12.61	0.2114	0	-1.3065	0.0618	-1.0333
4	-x, y+1/2, -z+1/2	13.1	-2.0083	-0.222	-1.6549	0.0618	-3.8234

N – number of molecules in contact; Symop – symmetry operation; R – centroid distance between molecules; E_ele – electrostatic interaction energy, E_pol - polarization interaction energy, E_dis - dispersion interaction energy, E_rep - repulsive interaction energy, E_tot - total interaction energy.

Table S5. Molecular pairs interaction energies (kJ/mol) for **Do5OH**.

N	Symop	R	E_ele	E_pol	E_dis	E_rep	E_tot
2	x, y, z	4.71	-16.701	-2.368	-73.4253	29.8494	-62.6445
4	x+1/2, y+1/2, -z+1/2	12.67	-48.516	-7.548	-17.0716	38.3778	-34.7581
2	-x, -y, -z	7.07	-9.513	-1.258	-40.1531	18.7872	-32.1369

2	-x, -y, -z	8.84	1.1627	-0.518	-12.0198	2.2866	-9.0885
4	-x+1/2, -y+1/2, z+1/2	14.40	1.2684	-0.222	-2.613	0.2472	-1.3194
2	-x, -y, -z	8.14	-7.7161	-1.48	-16.1135	7.8486	-17.461

N – number of molecules in contact; Symop – symmetry operation; R – centroid distance between molecules; E_ele – electrostatic interaction energy, E_pol - polarization interaction energy, E_dis - dispersion interaction energy, E_rep - repulsive interaction energy, E_tot - total interaction energy.

Table S6. Molecular pairs interaction energies (kJ/mol) for **Do6OH I**.

N	Symop	R	E_ele	E_pol	E_dis	E_rep	E_tot
2	-	6.99	-60.5661	-10.878	-27.9591	49.8108	-49.5924
2	-	7.17	-11.8384	-1.702	-31.6173	16.3152	-28.8425
4	-x, y+1/2, -z+1/2	10.89	-7.8218	-1.036	-16.8103	5.3766	-20.2915
2	-	11.85	-47.7764	-7.548	-10.2778	39.4902	-26.112

2	x, y, z	7.54	-22.0913	-2.294	-74.2963	35.226	-63.4556

N – number of molecules in contact; Symop – symmetry operation; R – centroid distance between molecules; E_ele – electrostatic interaction energy, E_pol - polarization interaction energy, E_dis - dispersion interaction energy, E_rep - repulsive interaction energy, E_tot - total interaction energy.

Table S7. Molecular pairs interaction energies (kJ/mol) for **Do6OH II**.

N	Symop	R	E_ele	E_pol	E_dis	E_rep	E_tot
4	x+1/2, -y+1/2, -z	10.00	-3.8052	-0.74	-8.9713	4.326	-9.1905
4	x+1/2, -y+1/2, -z	7.56	-3.171	-0.74	-28.1333	10.506	-21.5383
4	-x, y+1/2, -z+1/2	15.79	1.2684	-0.148	-2.1775	0.1236	-0.9335
2	x, y, z	4.63	-16.595	-2.294	-73.8608	29.8494	-62.9003
4	-x+1/2, -y, z+1/2	13.47	-55.175	-8.214	-15.9393	45.3612	-33.9675

N – number of molecules in contact; Symop – symmetry operation; R – centroid distance between molecules; E_ele – electrostatic interaction energy, E_pol - polarization interaction energy, E_dis - dispersion interaction energy, E_rep - repulsive interaction energy, E_tot - total interaction energy.
